# Influence of the synthesis method on the catalytic activity of mayenite for the oxidation of gas-phase trichloroethylene

**DOI:** 10.1038/s41598-018-36708-2

**Published:** 2019-01-23

**Authors:** Adriano Intiso, Joaquin Martinez-Triguero, Raffaele Cucciniello, Federico Rossi, Antonio Eduardo Palomares

**Affiliations:** 1 Department of Chemistry and Biology, University of Salerno, via Giovanni Paolo II, 132–84084 Fisciano, SA Italy; 2 Instituto de Tecnología Química, Universitat Politècnica de València-CSIC, Valencia, 46022 Spain; 3 Present Address: Department of Earth, Environmental and Physical Sciences - DEEP Sciences, University of Siena, Pian dei Mantellini 44, 53100 Siena, Italy

## Abstract

Catalytic oxidation of trichloroethylene (TCE) in heterogeneous phase (gas-solid) is an effective strategy for the conversion of this pollutant in less harmful compounds, namely CO_2_, CO and HCl. In this work, we have studied the use of mayenite, a cost-effective material, as an active catalyst for the TCE conversion. In particular, we have assessed the influence of the mayenite synthesis method (hydrothermal, sol-gel and ceramic) on the reaction performance. The materials have been characterized by different techniques, such as XRD, N_2_-sorption (BET), TPR, Raman spectroscopy, FESEM-EDX and TEM. The analysis of the light-off curves and product distribution, has shown that the use of the hydrothermal method for the mayenite synthesis results in the most active and selective catalyst. This has been related with a higher surface area and with a higher concentration of oxygen anions in the mayenite prepared by this method. It has been found that the presence of water in the stream do not influence the catalytic performance of the material. A mechanism for the reaction and for the partial deactivation of the catalyst has been proposed.

## Introduction

Trichloroethylene (TCE) is a common chlorinated volatile organic compound (VOC), widely employed until the eighties as organic solvent in industrial dry-cleaning applications and in painting and coating manufactures^1^. TCE can be classified either as a VOC or as a DNAPL (Dense Non-Aqueous Phase Liquid), depending on the context. TCE physico-chemical properties as its relatively low solubility, its high density and its low biodegradability, make TCE removal a scientific and technological challenge^2,3^.

Recently, the International Agency for Research on Cancer (IARC) reclassified TCE from “potential carcinogen” to “carcinogen”^4^. This, together with the widespread pollution in different environmental matrices^5^ raised several concerns in the public opinion and boosted the research of different remediation strategies as air stripping, adsorption, pump and treat, phytoremediation or thermal treatments^6–9^.

Thermal incineration has become the most common method to convert TCE in less harmful compounds, however, this method requires high costs related to the operative temperature (often higher than 1000 °C) and could form dangerous chlorinated by-products^10^. The use of heterogeneous (solid-gas) catalysts for this reaction might offer an effective alternative to overcome such drawbacks.

In the last years, different catalysts have been studied for the trichloroethylene oxidation^11–14^. Metal oxides and supported noble metals have been described as active catalysts, but they might promote the formation of toxic by-products, such as chlorine and perchloroethylene (PCE)^15^. Acid zeolites have also been tested, showing a good efficiency but suffering a deactivation process due to coke deposition and chlorine attack to the active sites^16^. Combination of transition metals and zeolites has been proposed to improve the activity and the selectivity towards less harmful products. Divakar *et al*.^17^, in particular, showed a good catalytic activity for beta and ZSM-5 zeolites doped with Fe. The catalytic performance of these materials depended on the distribution of the Fe-monomeric active species and this was related with the catalyst preparation method. Similarly, Blanch-Raga *et al*.^18^ tested Cu and Co doped beta zeolites in this reaction concluding that Cu-zeolite prepared by ion exchange was the best catalyst in terms of activity and selectivity, due to the combination of the zeolite acidic properties with the metal redox properties.

Rodríguez-Castellón and co-workers^19^ have investigated the influence of the catalyst surface area in the oxidation of organic molecules (*e.g*. propane). They prepared several catalysts (CeO_2_ and CuO-CeO_2_) by using different methods and they found that the catalyst surface area and the number of bulk/sub-surface defects were the main features to enhance the catalytic performance.

Recently^20,21^, we reported the use of mayenite (Ca_12_Al_14_O_33_) for the total oxidative conversion of TCE at temperatures as low as 450 °C (GHSV = 6000 h^−1^). The high activity of the catalyst was connected to the presence of O^2−^ and O_2_^2−^ anions in the micro-cages of the mayenite, which favoured the oxidation of TCE and avoided the formation of coke^22–25^. Mayenite presents several advantages compared with zeolites or noble and transition metals-based catalysts. It is an Earth-abundant and low-cost material that can be easily synthesized by different routes. In this work we evaluate the influence of the mayenite synthesis method on the catalyst activity and we study the reaction mechanism and the catalyst deactivation.

## Experimental

### Catalyst preparation

Mayenite was synthesized by different methods, namely hydrothermal, sol-gel and ceramic routes. Hydrothermal and ceramic mayenite were prepared by following the methods described by Li *et al*.^26^. In the hydrothermal synthesis a stoichiometric mixture of 41.5 g of Ca(OH)_2_ and 50.7 g of Al(OH)_3_ was added to 1 L of distilled water. The mixture was grounded to powder under magnetic stirring for 4 h at room temperature, afterwards it was placed in a stainless-steel autoclave at 150 °C for 5 h. The obtained solid was filtrated and dried at 120 °C overnight, crushed into fine powder and placed into a furnace at 600 °C in air for 4 hours. Ceramic mayenite was prepared by mixing powders of Ca(OH)_2_ and Al(OH)_3_ in stoichiometric ratio, grinding the mixture under magnetic stirring with distilled water for 4 h at 300 rpm. Afterwards the solid was dried at 120 °C overnight and calcined at 1000 °C for 4 h in air.

Sol-gel mayenite was prepared according to the procedure proposed by Ude *et al*.^27^ as follows: 69.6 g of calcium nitrate tetrahydrate, Ca(NO_3_)_2_ ∙ 4H_2_O, and 111.6 g of Al(NO_3_)_3_ ∙ 9H_2_O were dissolved in 1.5 L of distilled water. The mixture was heated at 60 °C and 5 g of citric acid, C_6_H_8_O_7_, were added. The citrate-nitrate mixture was heated and vigorously stirred at 90 °C until a gel was formed (about 12 h). The resulting gel was placed in a drying oven at 130 °C until a cake-like structure was obtained. The solid was then crushed into fine powder and finally placed into a furnace at 1000 °C in air for 4 h.

All catalysts were pelletized, and then the pellets were crushed and sieved to obtain grains of 0.25–0.45 mm in diameter. Materials were named as Maye Z, where Z corresponds to the catalyst preparation method, i.e. HA (hydrothermal), SG (sol-gel) and CR (ceramic).

### Catalyst characterization

Powder X-ray diffraction patterns (XRD) were collected by using an X’Pert-Pro diffractometer (Panalytical) equipped with an X’Celerator detector and using Ni-filtered Cu K radiation.

The surface area of the different catalysts was measured with an ASAP 2010 instrument (Micromeritics) using the BET method for the nitrogen adsorption isotherms at −196 °C.

Temperature programmed reduction (TPR) experiments were carried out using a TPD-TPR Autochem 2910 analyser equipped with a thermal conductivity detector. The reduction of the samples (10–20 mg) was conducted in the interval 25–800 °C with a thermal ramp of 10 °C min^−1^ using a N_2_:H_2_ flow (10% H_2_) of 50 mL min^−1^.

Field emission scanning electron microscopy images (FESEM) were obtained in a Zeiss Ultra 55 microscopy and the elemental analysis of the sample was carried out with energy-dispersive X-ray spectroscopy (EDX) in an INCA, Oxford Instrument.

High resolution transmission electron microscopy (HRTEM) images were obtained with a JEM2100F of 200 kV in HRTEM mode.

Raman spectra were recorded at RT with a 514 nm laser excitation by using a Renishaw Raman Spectrometer (“in via”) equipped with a CCD detector. The laser power on the sample was 25 mW and a total of 20 acquisitions were taken for each spectrum.

### Catalyst activity

The catalytic tests have been performed in a quartz fixed bed reactor. The desired mass of the catalyst (0.7 g), with 0.25–0.59 mm particle size, was located on a quartz plug located inside the reactor. Silicon carbide (>0.6 mm) was placed above the catalyst as a preheating zone. The temperature was measured with a K-thermocouple placed inside the reactor and the reactor was heated using an electrical oven.

The gaseous mixture was prepared saturating an air stream with liquid TCE at a controlled temperature. The reaction flow was composed by trichloroethylene (1000 ppm) and air with a gas flow of 400 mL/min (Gas Hourly Space Velocity, GHSV = 12.000 h^−1^). The residence time, based on the packing volume of the catalyst, was 0.24 s.

The reaction temperature was increased from 150 to 550 °C in steps of 50 °C. Each temperature was kept for 30 min and the overall length of the reaction was six hours. Wet experiments were performed by injecting water in the air flow with a syringe pump in order to have 1.7% of H_2_O in the gas flow.

The organic compounds of the gas flow were analysed with a Varian 3630 gas chromatograph equipped with a HP-5 column and with a flame ionization detector. CO and CO_2_ were separated with micro-packed columns and analysed with a thermal conductivity detector. Cl_2_ and HCl were absorbed in a solution containing NaOH 0.0125 M^28^.

The concentration of the absorbed Cl_2_ was determined by titration and the HCl concentration was measured by using an ion selective electrode. Blank experiments were made in the same conditions but without the presence of the catalyst. All the experiments were repeated three times to assure the reproducibility of the results. The error associated with the triplicate measurements was under 5%.

## Results and Discussion

### Catalyst characterization

**Figure 1:**
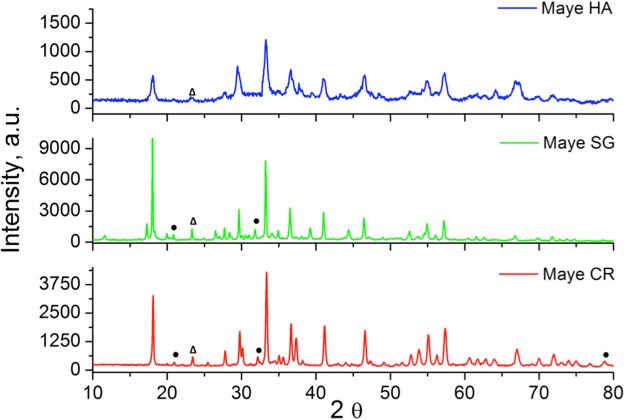
XRD patterns of synthesized catalysts: Hydrothermal mayenite (HA), Sol-gel Mayenite (SG), Ceramic Mayenite (CR).

Figure 1 shows the XRD patterns of the catalysts that have been synthesized by the different methods. The catalysts’ crystalline structure was indexed within I-43d group. All the XRD patterns showed the characteristic peaks of mayenite (Ca_12_Al_14_O_33_) at 2θ = 18.1°, 30°, 33.4°, 36.7°, 41.2°, 46.7°, 55.2° and 57.4°. Ca_3_Al_2_O_6_ (•) and CaAl_2_O_4_ (Δ) were also detected in traces as usual impurities formed during the mayenite preparation process^23^.

Comparing the XRD patterns, it can be observed that the mayenite synthesized by the hydrothermal process (Maye HA) showed broader and less intense peaks with respect to the other samples. As the width of the diffraction peak is inversely related with the crystalite size^29^, the results indicate that Maye HA is less crystalline with a smaller crystallite size.

XRD analysis is consistent with TEM micrographies of Maye HA and Maye SG (See the supporting material Fig. S3). TEM images clearly showed that the crystallite size of the sample prepared by the sol-gel method is bigger than the crystallite size of the sample prepared by the hydrothermal method.

Table 1 reports the BET surface area and the H_2_ uptake in the TPR experiments for the different synthesized mayenites. Mayenite prepared by the hydrothermal route showed a significantly higher BET surface area (~3 times larger) compared to the others, whilst ceramic mayenite had the lowest one. These results are consistent with those obtained in the XRD study, *i.e*. the broadest peaks of maye HA due to a lower crystallite size, result in a higher external surface area and then in a higher BET surface area.

**Table 1:** BET surface area and H_2_ uptake during TPR for the different mayenites.

Catalyst	BET surface area (m^2^/g)	H_2_-uptake (mmol H_2_/g)
Maye HA	35.5	1.19
Maye SG	14.9	1.16
Maye CR	11.7	0.45

**Figure 2:**
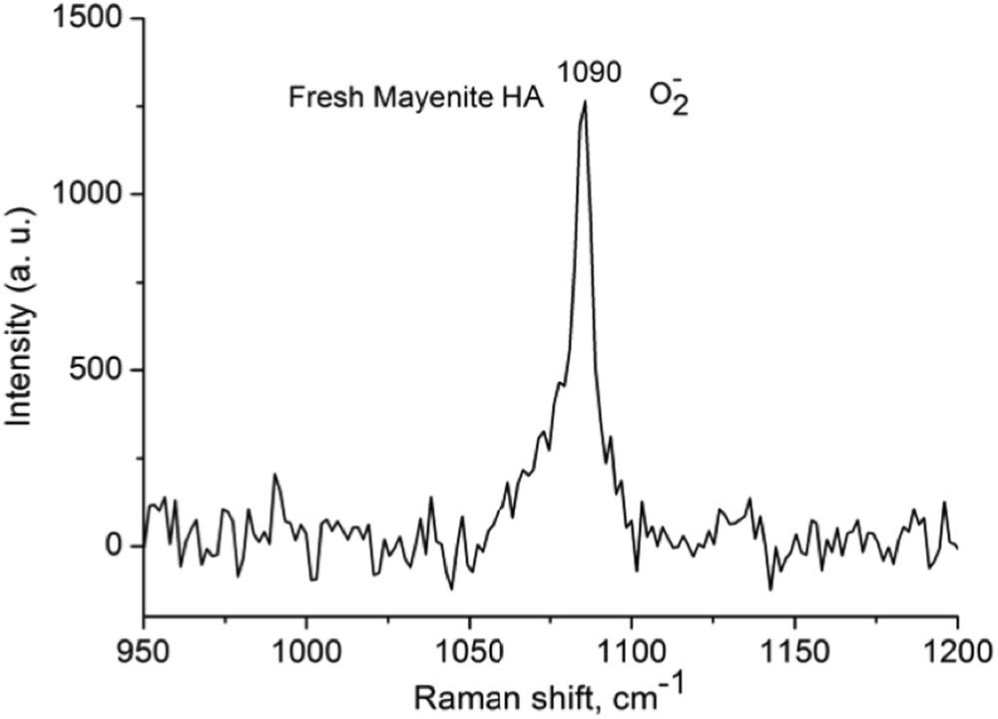
Raman spectra of fresh HA mayenite.

Figure 2 shows the Raman spectrum of the maye HA. The only band observed appears at 1090 cm^−1^ that, according to literature, is assigned to superoxide radicals O_2_^−^^25^.

**Figure 3:**
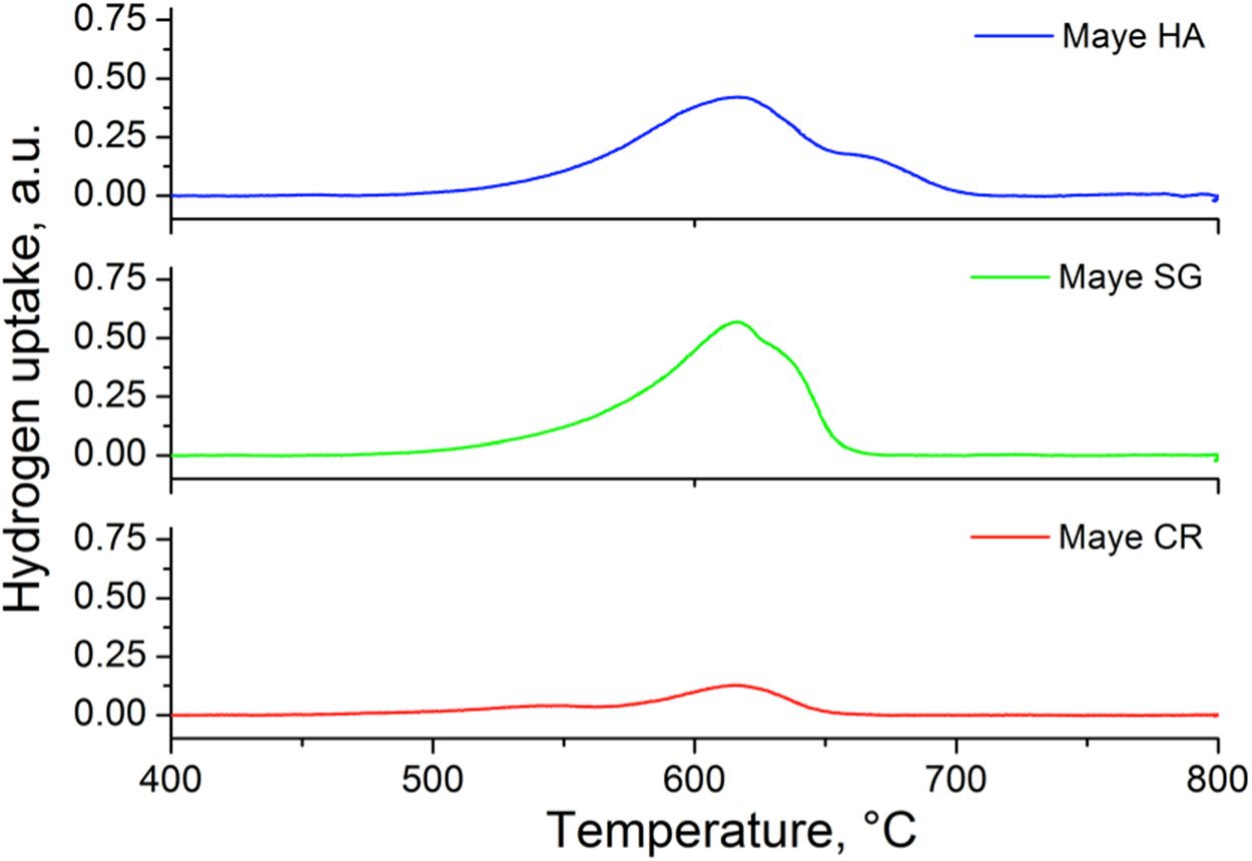
TPR profiles of the different synthesized mayenites.

Figure 3 presents the H_2_-TPR profiles of the mayenites tested. It can be seen that for all the samples, the hydrogen consumption starts around 550–575 °C. At this temperature most of the mayenite surface is free of hydroxyl groups^30^ and H_2_ can be adsorbed on the Al_3c_ and O^2−^_3c_ Lewis acid-base pairs that, in turn, can dissociate it in a heterolitic fashion.

Increasing the temperature, the dissociatively adsorbed H_2_ can penetrate into the cages to react with extra framework O^−^_x_ and O_2_^2−^ anions^30^, with an observed maximum of H_2_ consumption around 620 °C. A shoulder at higher temperature (630 °C) is observed in the Maye SG and an incipient new band appears at 660 °C in Maye HA suggesting the presence of other reducible species in these samples. The hydrogen uptake reported in Table 1 shows that Maye HA has the same hydrogen consumption of Maye SG (around 1.1 mmol/g), being this value double than that obtained with Maye CR (0.45 mol/g). According to the literature^30^ the complete reduction of extra-framework anions in the mayenite requires a consumption of about 1 mmol H_2_/g. Comparing this value with those shown in Table 1 it can be stated that all guest oxygen anions in Maye HA and Maye SG were completely reduced after the TPR, but this did not occur for the Maye CR. These results indicate a higher population of reducible species in the Maye HA and in Maye SG than in Maye CR; however, while in Maye HA reducible species were identified as O_2_^−^ anions, in maye CR many impurities of NO_2_^−^ and NO_3_^−^ are present due to the precursor employed in the synthesis^31^. A higher concentration of oxygen species in Maye HA is likely due to the lower calcination temperatures and to a higher number of nanocages, able to entrap active oxygen anion species unstable in the atmosphere^26^.

### Catalytic activity

**Figure 4:**
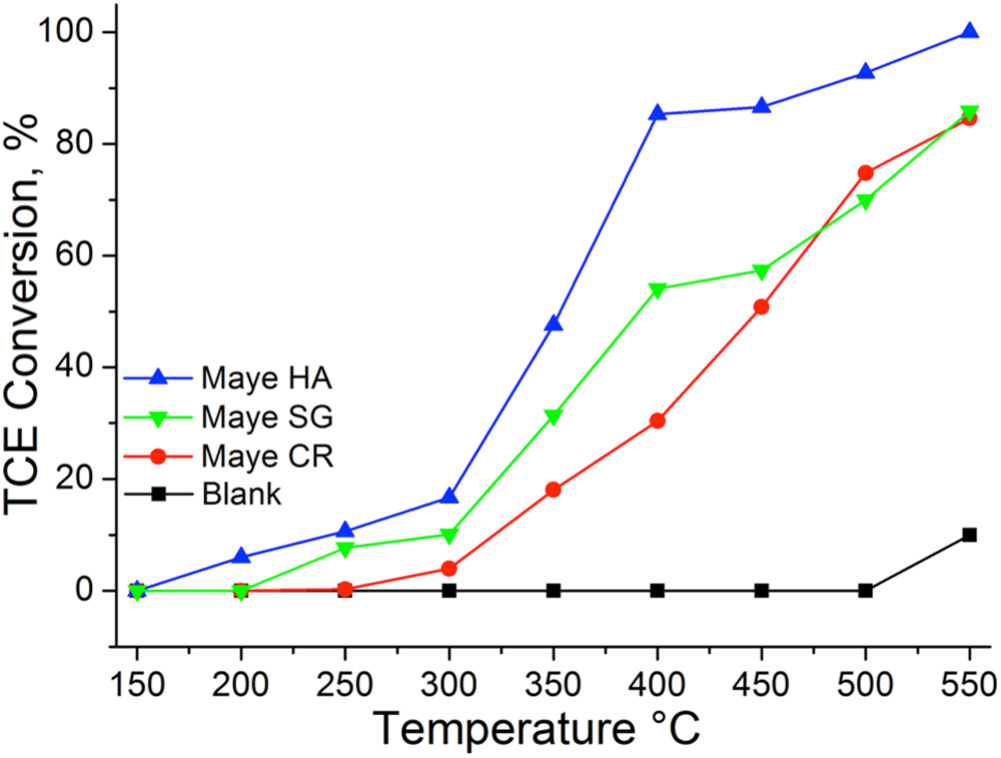
TCE conversion in dry conditions for mayenites synthesized with different methods. A blank experiment (thermal oxidation) is also shown.

The catalyst performance, in the trichloroethylene oxidation, of the different mayenites was evaluated by monitoring the TCE conversion as a function of the temperature (light-off curve). In Fig. 4 are reported the conversion curves for a blank experiment (no catalyst in the reactor), for Maye HA, Maye SG and for Maye CR in dry conditions. The conversion percentage was calculated as the ratio between the reacted TCE over the total TCE delivered into the reactor

1 $${\rm{TCE}}\,{\rm{conversion}}\,( \% )=\frac{{m}_{TCE}^{i}-{m}_{TCE}^{o}}{{m}_{TCE}^{i}}\times 100$$

where ${m}_{TCE}^{i}$ are the moles of TCE delivered into the reactor and ${m}_{TCE}^{o}$ are the number of moles measured at the reactor outlet.

Figure 4 shows that there was no conversion below 500 °C in the absence of the catalyst (thermal oxidation), whereas in the presence of mayenite, the TCE oxidation started around 150–250 °C, depending on the synthetic route. Mayenite prepared by the hydrothermal method showed the best performance among the different materials. The T_50%_ (temperature at which 50% of the trichloroethylene conversion was reached) was around 350 °C for Maye HA, whilst for Maye SG and Maye CR was 390 °C and 450 °C, respectively. The conversion yield increased with the temperature, until a complete conversion was reached at 550 °C for Maye HA whilst at this temperature Maye SG and Maye CR only converted ~85% of TCE. Table 2 resumes the performances of the different materials. The results obtained with Maye HA showed a better catalytic performance compared to other catalysts described in the literature as active catalysts for this reaction as beta zeolites and Co-doped beta zeolites^18^ and is in line with the performance of Fe-doped ZSM-5 zeolites^14^.

**Table 2:** Activity of the different samples of mayenite with respect to those reported in literature.

Catalyst	T_10_ (°C)	T_50_ (°C)	T_90_ (°C)
Maye HA^a^	245	350	490
Maye SG^a^	300	390	>550
Maye CR^a^	320	450	>550
Blank	550	>550	>550
*Co-beta zeolite* ^b,^ ^18^	250	400	>550
*Fe-ZSM5 zeolite* ^c,^ ^14^	300	390	460

T_10_, T_50_ and T_90_ are the temperature at which 10, 50% and 90% of TCE was converted, respectively. ^a^GHSV = 12000 h^−1^; ^b^15000 h^−1^; ^c^13500 h^−1^. ^a,b,c^[TCE] = 1000 ppm.

The results obtained also show that, although all the mayenites have the same chemical composition, there are important differences in their catalytic activities. This must be related to different physico-chemical properties, being the most important differences those related with the surface area and the hydrogen uptake in the TPR experiments. Table 1 shows that mayenites prepared by hydrothermal route and by the sol-gel process have the highest hydrogen uptake (around 1.1 mmol H_2_ g^−1^), this implies a highest amount of reducible species. Raman spectra have shown that these species are superoxide radicals O_2_^−^ that are reduced in the TPR experiments. It has been described that these species are the active sites for the TCE oxidation^20^, then we can infer that the samples with a larger amount of oxygen radicals, i.e. Maye HA and Maye SG, must show a higher activity. Nevertheless, mayenite prepared by the hydrothermal route is more active than the mayenite prepared by the sol-gel process, although both catalysts have a similar hydrogen consumption (Table 1). Then, the different activity of both materials should be related with their different surface area (see Table 1), obtaining better results with the material with the highest surface area, this is the Maye HT, which allows a better accessibility of TCE to the active sites. Then we can conclude that both, surface area and the presence of ionic oxygen species, are necessary for the catalytic performance of mayenite and an adequate combination of high surface area and high number of active species will result in a better activity of the mayenite for the TCE oxidation. In this system the role of the O_2_ present in the reaction feed is the regeneration of the active oxygen species (O^2−^, O^−^, O_2_^−^) of the catalyst surface that in turn, can react and oxidize TCE^32^. Contrarily to what it occurs with other catalysts used in this reaction, e.g. zeolites^11^, acidity does not play an important role in the TCE oxidation when using this type of materials.

The effect of water vapour on the TCE catalytic oxidation was also investigated. The results showed that the addition of water to the feed stream did not alter significantly the activity of the materials (details in the Supporting Material, Fig. S4).

### Product distribution

For all the samples prepared, the main oxidation products of the trichloroethylene oxidation reaction were found to be carbon dioxide, carbon monoxide and hydrogen chloride. More harmful by-products, such as Cl_2_ and PCE, were detected only in traces at the different temperatures investigated. In contrast to other catalysts like zeolites^18^, the scarce formation of PCE in mayenites is due to the absence of H-Brönsted acid sites, necessary for the PCE formation.

The selectivity for CO and CO_2_ (S_CO_ and ${{\rm{S}}}_{{{\rm{CO}}}_{2}}$) was calculated from equations (2) and (3), where CO and CO_2_ are the products’ concentration expressed in ppm. As the formation of PCE was depreciable compared with the CO and CO_2_ it was not considered for the calculation of the selectivity.

2 $${S}_{{\rm{CO}}}=\,\frac{{\rm{CO}}}{(\mathrm{CO}+{{\rm{CO}}}_{{\rm{2}}})}\times 100$$

3 $${S}_{{{\rm{CO}}}_{{\rm{2}}}}=\frac{{{\rm{CO}}}_{{\rm{2}}}}{(\mathrm{CO}+{{\rm{CO}}}_{{\rm{2}}})}\times 100$$

As reported in Table 3, at 550 °C Maye HA showed a better selectivity towards CO_2_ than maye SG and maye CR, as expected from the highest accessibility of the reactants to the active sites in maye HA that favours the complete oxidation of the TCE carbon atoms.

**Table 3:** Carbon selectivity at 550 °C.

Catalyst	S_CO_ (%)	${{\bf{S}}}_{{{\bf{CO}}}_{{\bf{2}}}}$ (%)
Maye HA	30	70
Maye SG	45	55
Mare CR	60	40

Regarding to the selectivity of the different catalysts towards the chlorinated species, it was observed that HCl was the main product detected in the exhaust gases. The hydrogen atoms that are necessary to form the HCl molecule came both from the trichloroethylene molecules and from water impurities in the gas stream. Small traces of Cl_2_ were noticed and no other Cl-compounds were detected in the gas phase.

In Table 4 is reported the selectivity to HCl of the different catalysts studied. The selectivity has been calculated (eq. 4) as the moles of HCl formed related with the moles of Cl reacted.

4 $${S}_{{\rm{HCl}}}=\frac{{\rm{HCl}}}{{{\rm{Cl}}}_{{\rm{reacted}}}}\times 100$$

**Table 4:** HCl selectivity at 550 °C.

Catalyst	S_HCl_ (%)
Maye HA	14
Maye SG	13
Maye CR	17

The results clearly show that an important part of the Cl atoms formed other species than HCl. These results only can be explained by a partial reaction of the mayenite with the HCl formed during the reaction resulting in the formation of chloromayenite (Brearleyite, Ca_12_Al_14_O_32_Cl_2_).

Similar results were obtained when the reaction was made in presence of water (1.7%), showing that the presence of moisture in the feeding stream did not affect the selectivity of the reaction.

### Catalyst Stability

**Figure 5:**
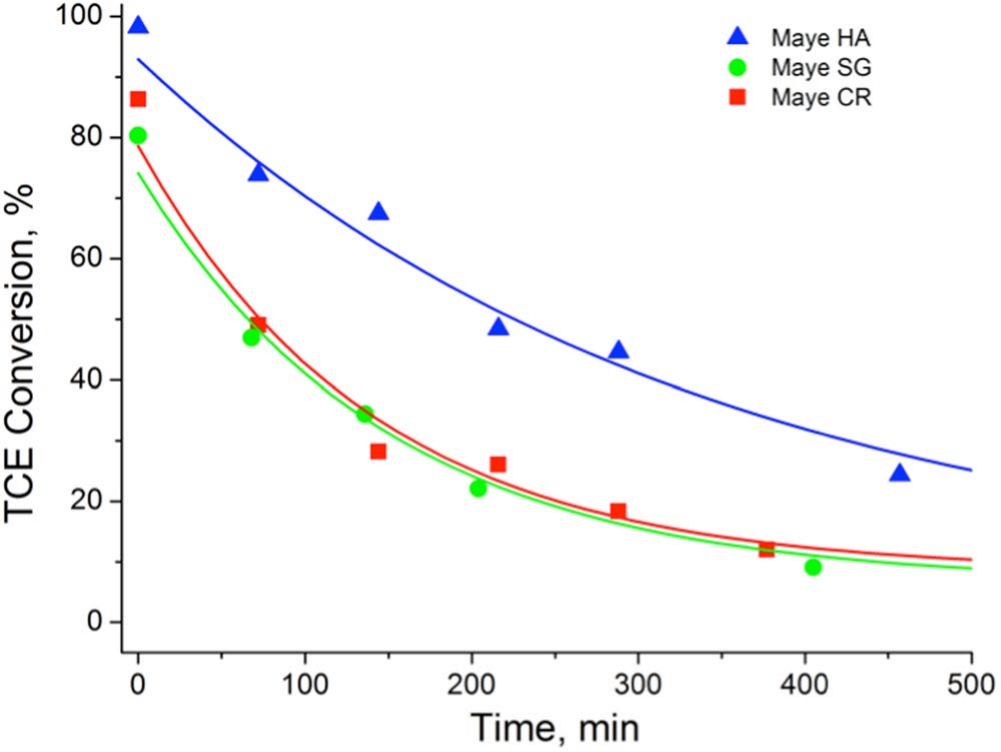
TCE conversion over mayenite samples at 500 °C for 500 min (catalyst = 0.7 g, [TCE] = 1000 ppm, flux = 400 mL/min, GHSV = 12000 h^−1^, T = 500 °C). Lines represent the exponential fitting of the experimental data.

The stability of the catalysts and the possible formation of chloromayenite were studied by a long-term experiment at 500 °C. The results are shown in Fig. 5.

As it can be seen, a moderate and progressive deactivation of the catalysts appeared with the three materials. To have a rough estimation of the deactivation kinetics, the experimental data were fitted with a simple exponential decay function having the form $y=A{e}^{-kt}+{y}_{0}$; the fitting procedure yielded a deactivation constants of *k*_HA_ = 3 × 10^−3^ min^−1^, *k*_SG_ = 6.7 × 10^−3^ min^−1^ and *k*_CR_ = 7.2 × 10^−3^ min^−1^. This indicates that a faster deactivation occurs with the mayenite prepared by the ceramic or sol-gel method, showing again a better catalytic performance of the mayenite prepared by hydrothermal process. The same results were obtained in presence of water indicating that, contrarily to what it occurs with other materials^11^, in this case water does not prevent catalyst deactivation.

**Figure 6:**
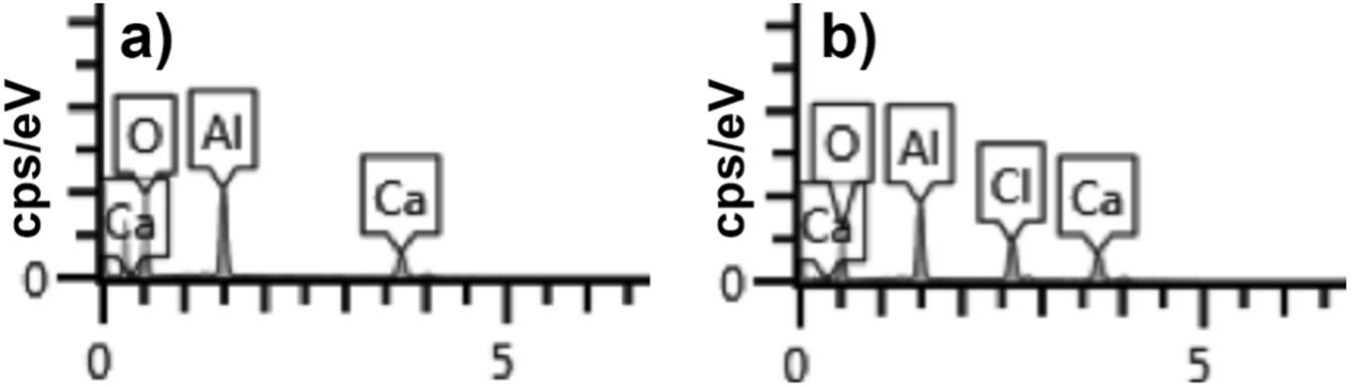
EDX spectra of mayenite HA (**a**) before and (**b**) after the oxidation reaction.

To understand the deactivation process a further characterization of the catalyst after reaction was performed. XRD patterns (see Supporting Material S1) of the catalyst before and after reaction do not show structural changes that could explain the deactivation of the catalyst. Nevertheless, the presence of a new peak at 38.4° related with chloromayenite suggests a reaction of the HCl formed during the reaction with the catalyst basic sites. FESEM-EDX images of mayenite were also recorded before and after the catalytic oxidation (see Supporting Material S2). It was observed that no significant morphological changes of the mayenite structure occurred after the oxidation reaction. Nevertheless an EDX analysis of the catalyst after reaction (Fig. 6), showed that a uniform distribution of chloride appeared on the mayenite surface, consistently with XRD experiments.

**Figure 7:**
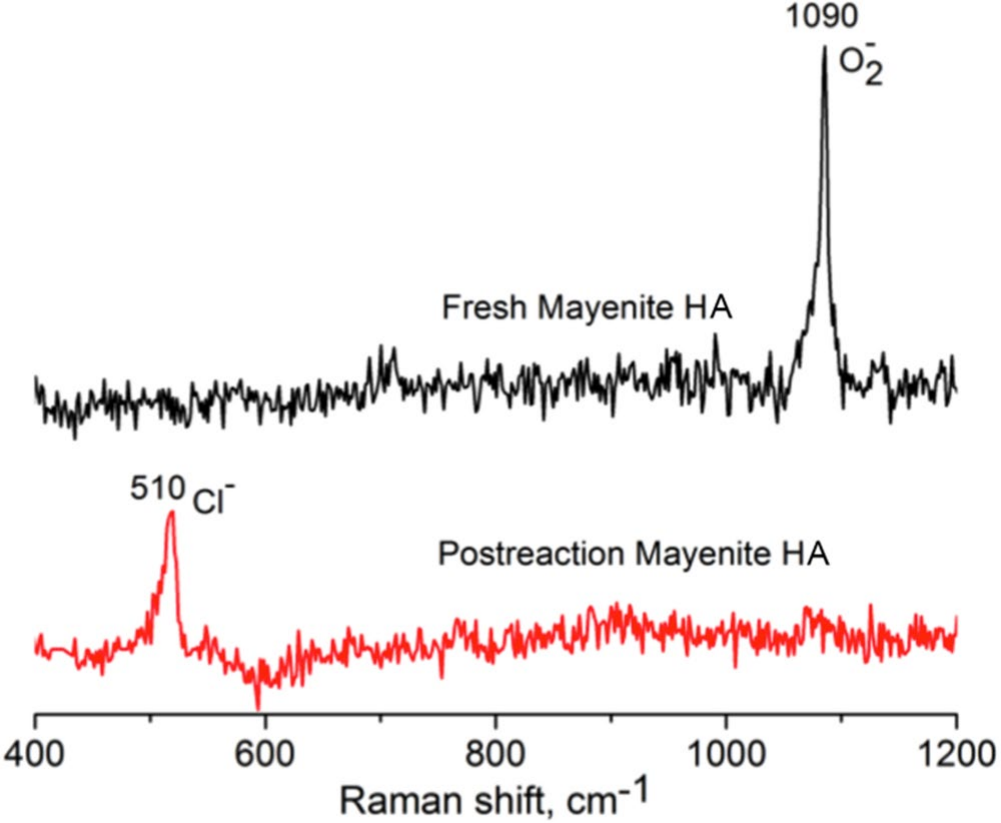
Raman spectra of HA mayenite fresh (top) and after reaction (bottom).

The formation of HCl during the reaction caused a partial irreversible substitution of the anionic oxygen species (O_x_^x−^) by chloride ions forming chloromayenite^33^, with the consequent deactivation of the catalyst. This was definitely proved by a characterization of the postreaction catalysts by means of Raman spectroscopy, a valuable tool to identify functional groups in the mayenite family minerals^34^. In fact, as shown in Fig. 7, the O_2_^−^ band at 1090 cm^−1^ observed in the fresh mayenite, disappeared after the long-term reaction, with the concomitant appearance of a new Cl^−^ band at 510 cm^−1^. The signal at 510 cnm^−1^ is also characteristic of the mineral chloromayenite^35^. Contrarily to what it occurs with other catalysts in this reaction, chloride ions react with the support forming a new stable phase (chloromayenite) and they can not react through the Deacon reaction to form Cl_2_ even at temperatures higher than 350 °C as it was evidenced by the absence on Cl_2_ in the exhaust gases.

The influence of chlorine in the mayenite deactivation process, was definitively proved by performing an experiment using a not-chlorinated VOC compound such as toluene (catalyst = 0.7 g, [Toluene] = 1000 ppm, flux = 400 mL/min, GHSV = 12000 h^−1^, T = 550 °C). The results showed an excellent stability of mayenite after 5 hours of reaction without any loss of activity and selectivity (details in Fig. S4 of the supporting material). Raman experiments made after this new reaction (Fig. S5 in the supporting material) showed the maintenance of the bands assigned to the active oxygen species at the end of the reaction. Then it can be confirmed that mayenite deactivation during the TCE oxidation was due to the chlorine anions, as also recently reported Hosono *et al*.^36^. Further works should be conducted to improve the stability of mayenite in TCE oxidation. In this respect, the use of metal-doped mayenite, for example with ruthenium^36^, seems to prevent or, at least, mitigate the halogen poisoning effects.

## Conclusions

Mayenite was prepared by different methods to evaluate the influence of the preparation method on the catalytic activity for TCE oxidation. The physico-chemical properties of the catalysts were determined by XRD, N_2_-sorption (BET), Raman spectroscopy, TPR and FESEM-EDX and correlated with the catalytic performance. The synthesized catalysts were active for the TCE oxidation, being CO_2_, CO and HCl the main reaction products and avoiding the formation of toxic organic by-products such as PCE. It has been also shown that the presence of moisture in the gas feed did not alter the activity neither the selectivity of the different catalysts. The mayenite prepared by the hydrothermal method (maye HA) showed a better catalytic performance compared with the mayenites synthesized by other routes. This is due to an optimum combination of surface area and redox active species, responsible for the catalytic activity of the material. Nevertheless, all the samples showed a progressive deactivation, due to the formation of chlorinated species that replaced the active anionic oxygen sites responsible for the TCE oxidation, being the maye HA the most stable one.

In conclusion, we demonstrated that the mayenite synthesis procedures influence the catalytic performance of the material in the TCE reaction, being necessary the use of a synthesis method that maximize the oxidative properties and the surface area of the mayenite.

## Electronic supplementary material

Supplementary Information
