# Supplementary material for: Influence of the synthesis method on the catalytic activity of mayenite for the oxidation of gas-phase trichloroethylene
Source: Sci Rep. 2019 Jan 23;9:425. doi: 10.1038/s41598-018-36708-2 (PMC6344594; doi:10.1038/s41598-018-36708-2)
Supplement: Supplementary file 1 — Supplementary Information [file 41598_2018_36708_MOESM1_ESM.docx]

**Supporting material**

**Influence of the synthesis method on the catalytic activity of mayenite for the oxidation of gas-phase trichloroethylene**

Adriano Intiso,^a^ Joaquin Martinez-Triguero,^b^ Raffaele Cucciniello,^a^ Federico Rossi^a†^* and Antonio Eduardo Palomares^b^*

^a^ Department of Chemistry and Biology, University of Salerno, via Giovanni Paolo II, 132 – 84084 – Fisciano (SA), Italy.

^b^ Instituto de Tecnología Química, Universitat Politècnica de València-CSIC, Valencia 46022, Spain

^†^Present Address: Department of Earth, Environmental and Physical Sciences - DEEP Sciences – University of Siena, Pian dei Mantellini 44, 53100 Siena - Italy

*Corresponding Author: FR [federico.rossi2@unisi.it](mailto:federico.rossi2@unisi.it); AEP [apalomar@iqn.upv.es](mailto:apalomar@iqn.upv.es)

**S1 Catalyst Stability**

**XRD spectra of hydrothermal mayenite**

XRD patterns of hydrothermal mayenite, before and after stability test, are reported in Fig. S1. Results show no changes in the crystalline structure [1], and the presence of a new peak at 38.4° is attributed to chloromayenite (Brearleyite, Ca_12_Al_14_O_32_Cl_2_) as reported in literature [2,3].


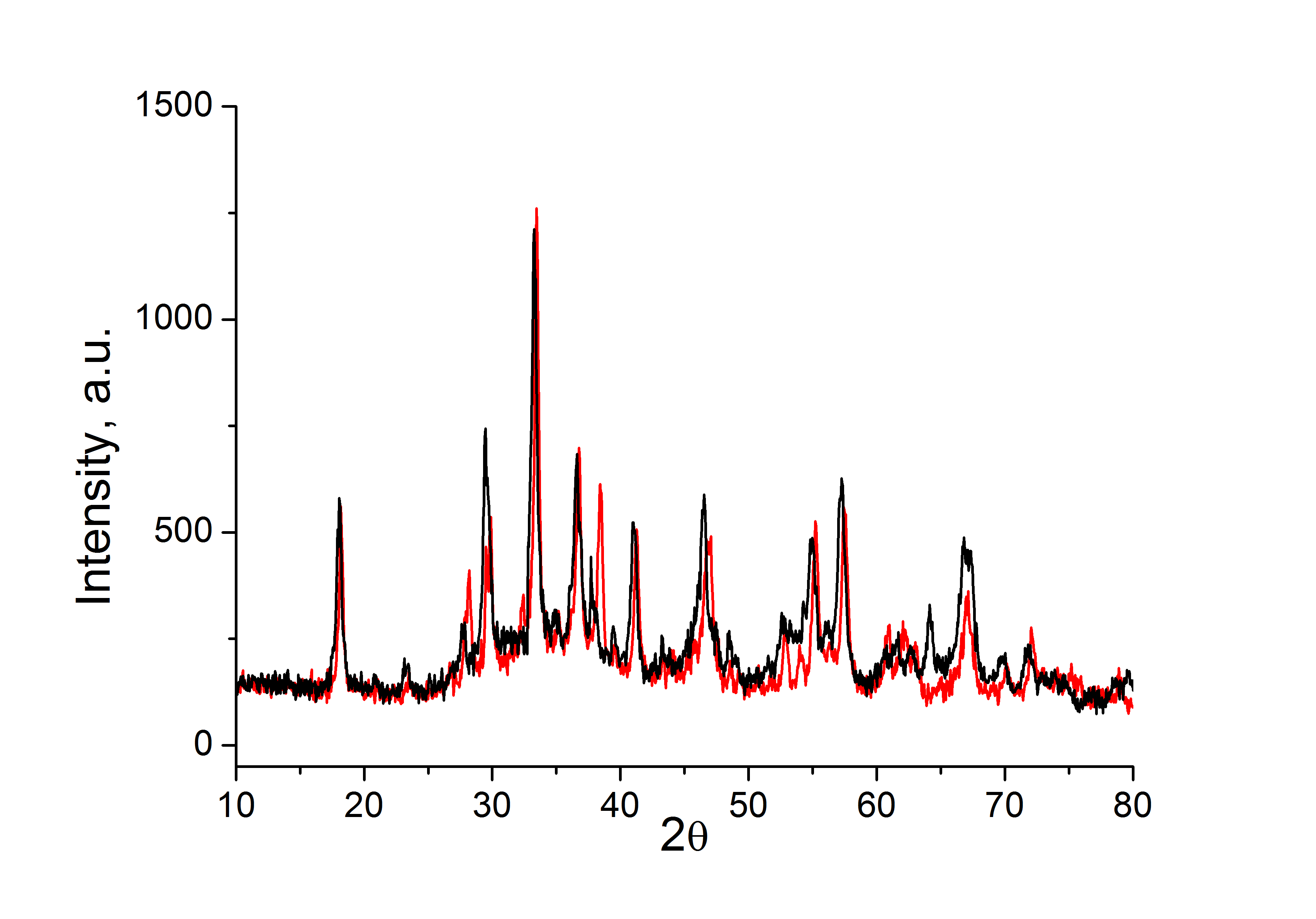


**Fig.** **S1.** XRD patterns of mayenite before (black line) and after (red line) stability test (maye HA = 0.7 g, [TCE] = 1000 ppm, flux = 400 mL/min, GHSV =12000 h^-1^, T = 500 °C).

**FESEM analysis of hydrothermal mayenite**

In Fig. S2 are reported the FESEM images of mayenite before (left) and after (right) stability test.





**Fig. S2.** FESEM images of mayenite before (left) and after (right) stability test

**TEM Images of the materials**

In Fig. S3 are reported the TEM images of mayenite synthetized by the sol-gel method (left) and by the hydrothermal method (right).


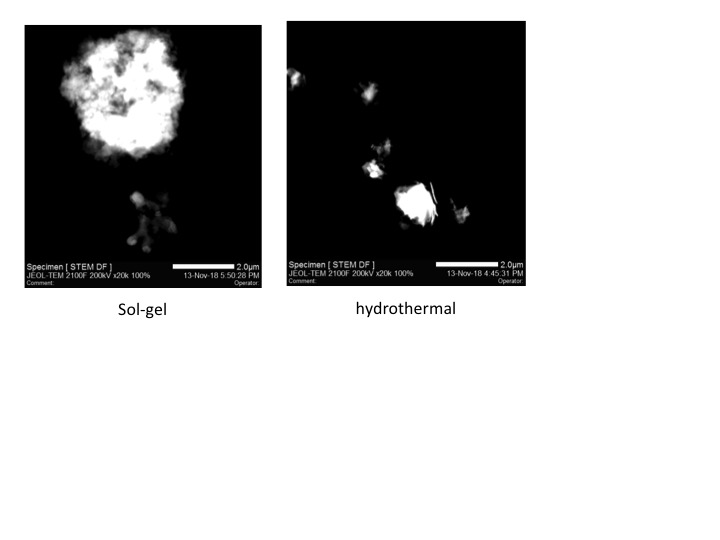


**Fig. S3.** TEM Images of mayenites

**S2 Influence of water on mayenite activity**

Figure S.3 reported the conversion percentage curves at different temperatures for Maye HA, Maye SG and Maye CR for wet conditions (1.7 %).


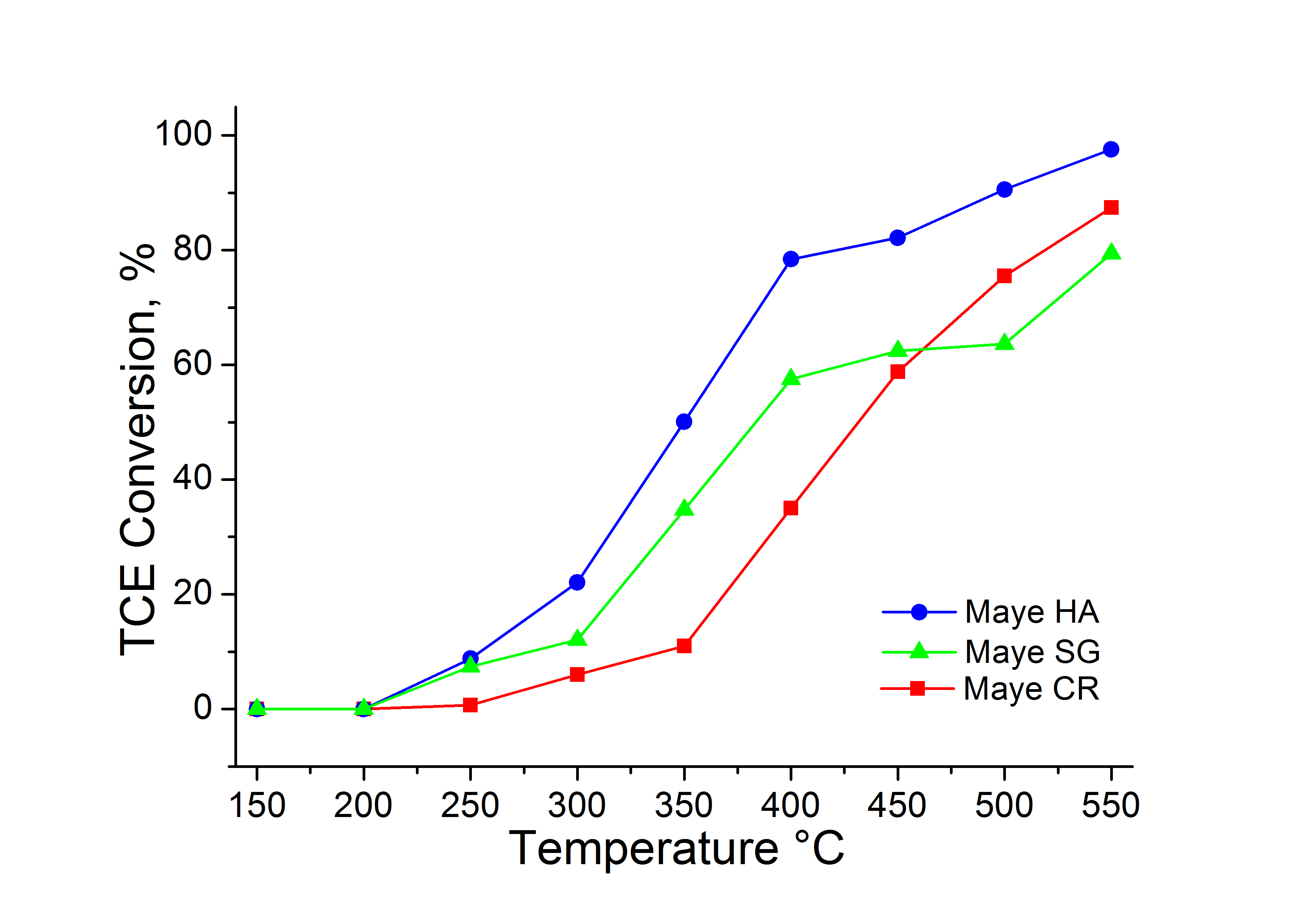


**Fig. S4.** TCE conversion in wet conditions (1.7%) for mayenite synthesized with different methods.

**S3 Toluene oxidation over mayenite catalyst**

**
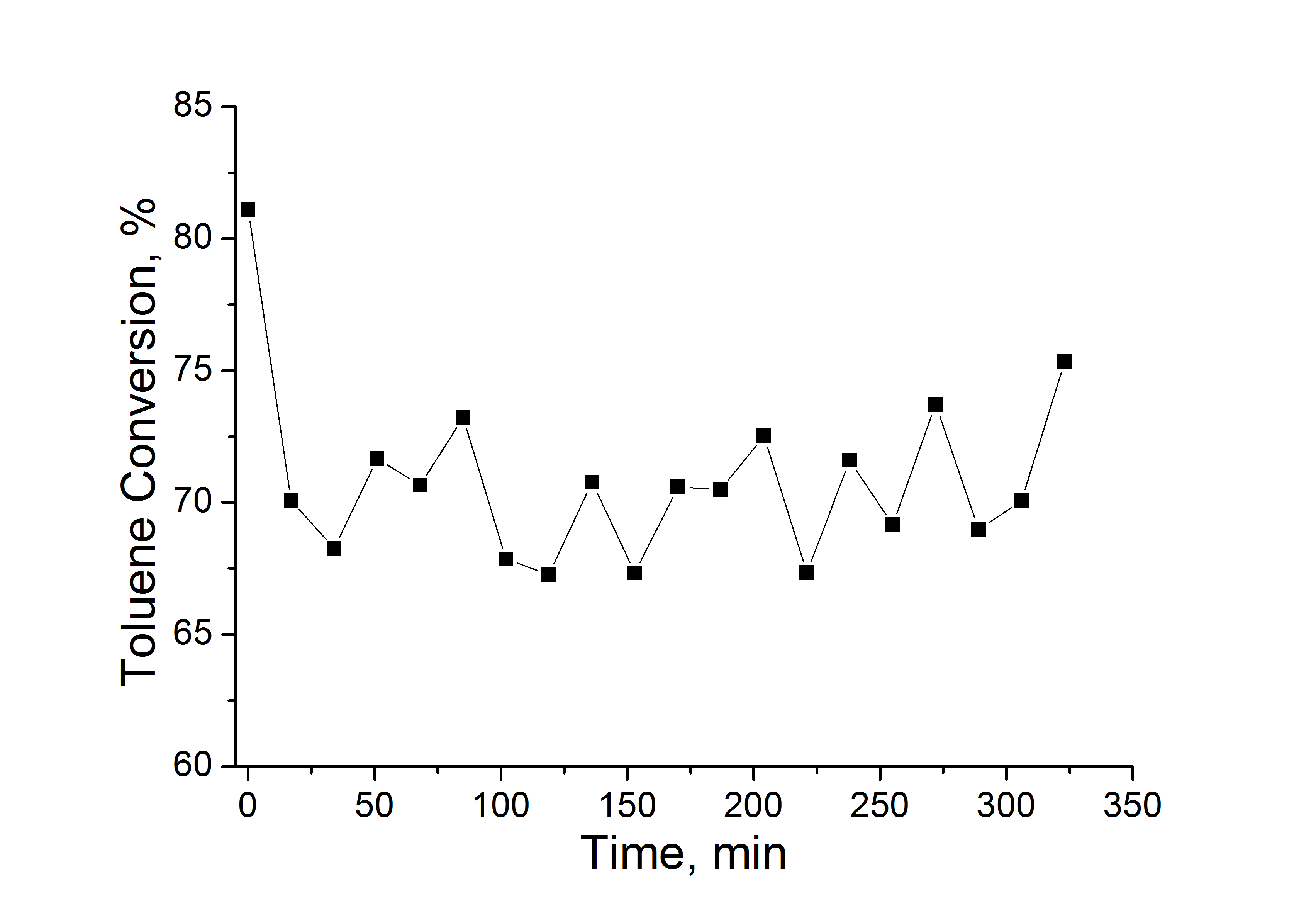
**

**Fig. S5.** Toluene conversion over maye HA at 550 °C for 350 min (catalyst = 0.7 g, [Toluene] = 1000 ppm, flux = 400 mL/min, GHSV =12000 h^-1^, T = 550 °C).


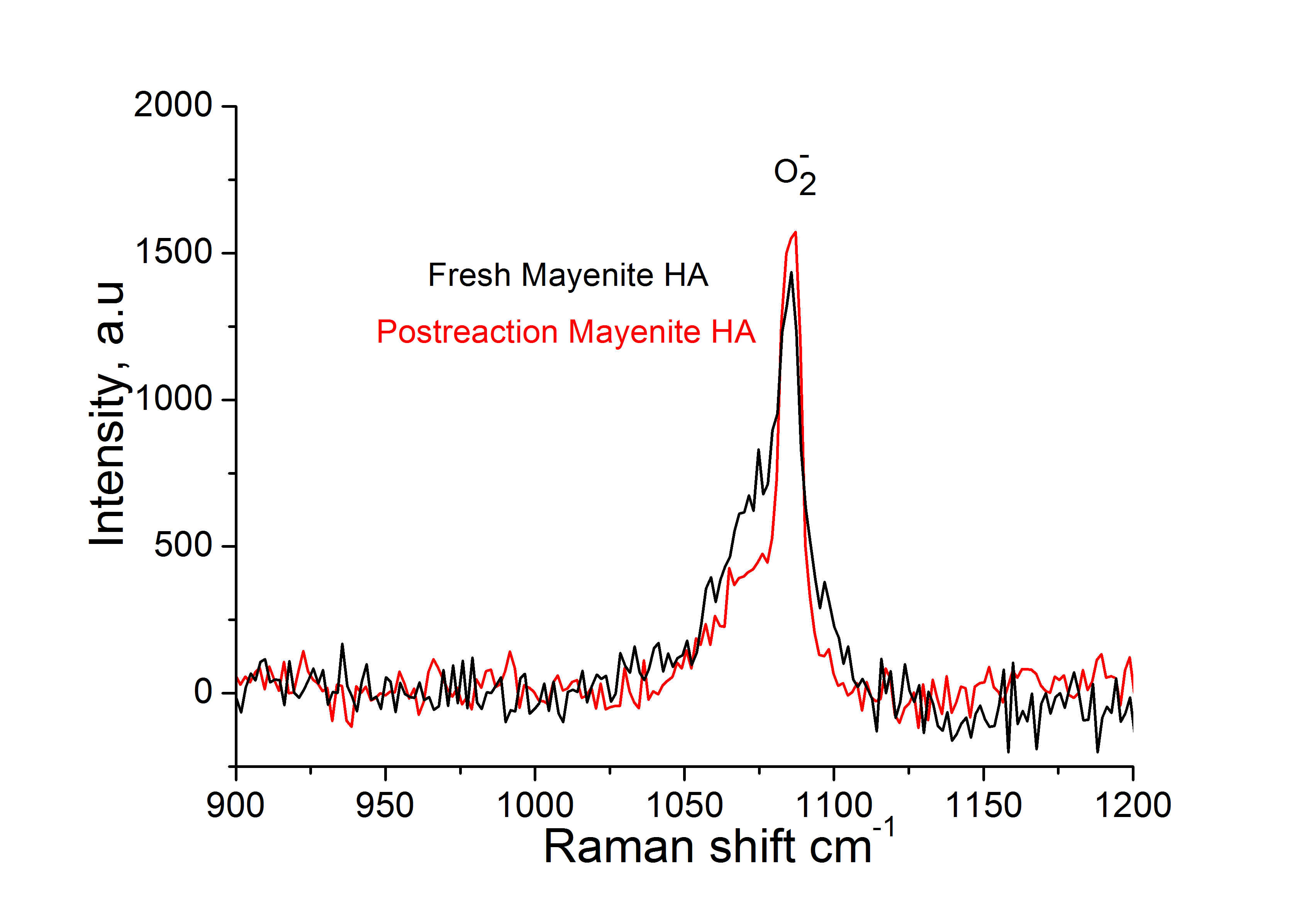


**Fig. S6.** Raman spectra of HT mayenite fresh (black) and after reaction (red) with toluene.

**References**

[1] J. Li, M. Kitano, T.-N. Ye, M. Sasase, T. Yokoyama, H. Hosono, ChemCatChem 9 (2017) 3078–3083.

[2] A. Schmidt, M. Lerch, J.-P. Eufinger, J. Janek, I. Tranca, M.M. Islam, T. Bredow, R. Dolle, H.D. Wiemöfer, H. Boysen, M. Hölzel, Solid State Ionics. 254 (2014) 48–58.

[3] R. Cucciniello, A. Intiso, S. Castiglione, Genga A, Proto A, Rossi F. App. Cat. B: Environ. 204 (2017) 167–172.
